# Myocardial Slices: an Intermediate Complexity Platform for Translational Cardiovascular Research

**DOI:** 10.1007/s10557-019-06853-5

**Published:** 2019-01-22

**Authors:** Samuel A. Watson, Cesare M. Terracciano, Filippo Perbellini

**Affiliations:** National Heart & Lung Institute, Imperial College London, London, UK

**Keywords:** Myocardial slice, Organotypic heart slices, Multicellularity, Cardiac model

## Abstract

Myocardial slices, also known as “cardiac tissue slices” or “organotypic heart slices,” are ultrathin (100–400 μm) slices of living adult ventricular myocardium prepared using a high-precision vibratome. They are a model of intermediate complexity as they retain the native multicellularity, architecture, and physiology of the heart, while their thinness ensures adequate oxygen and metabolic substrate diffusion in vitro. Myocardial slices can be produced from a variety of animal models and human biopsies, thus providing a representative human in vitro platform for translational cardiovascular research. In this review, we compare myocardial slices to other in vitro models and highlight some of the unique advantages provided by this platform. Additionally, we discuss the work performed in our laboratory to optimize myocardial slice preparation methodology, which resulted in highly viable myocardial slices from both large and small mammalian hearts with only 2–3% cardiomyocyte damage and preserved structure and function. Applications of myocardial slices span both basic and translational cardiovascular science. Our laboratory has utilized myocardial slices for the investigation of cardiac multicellularity, visualizing 3D collagen distribution and micro/macrovascular networks using tissue clearing protocols and investigating the effects of novel conductive biomaterials on cardiac physiology. Myocardial slices have been widely used for pharmacological testing. Finally, the current challenges and future directions for the technology are discussed.

Myocardial slices, also known as “cardiac tissue slices” or “organotypic heart slices,” are ultrathin slices of living adult ventricular myocardium prepared using a high-precision vibratome (Fig. 1A). They are described as models of intermediate complexity as they retain the native multicellularity, complex extracellular architecture (Fig. 1B/C), and physiology of adult cardiac tissue, while their thinness (100–400 μm) ensures adequate oxygen diffusion and the maintenance of viability in the absence of coronary perfusion in vitro [1]. Slices can be produced from human cardiac tissue, in addition to small and large mammalian hearts, thus providing a representative in vitro platform for translational cardiovascular research.

**Fig. 1:**
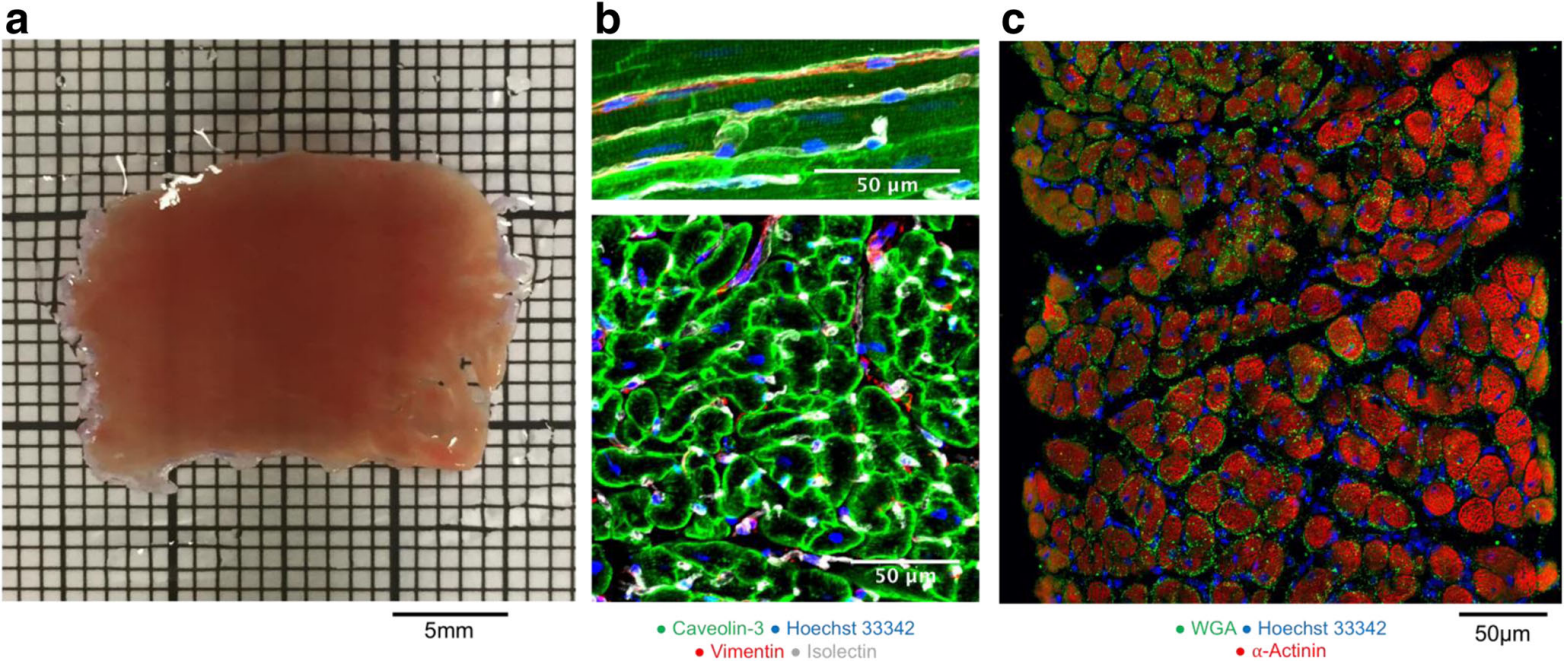
Myocardial slices—native multicellularity and intact architecture. A, freshly prepared adult rat myocardial slice place on a 1 × 1 mm grid. Image previously published in *Nature Protocols* [1] B, immunohistochemical staining and confocal microscopy were used to identify cardiac cells within a freshly prepared dog myocardial slice. Top, longitudinal section; bottom, transverse section. Cardiomyocytes were labeled with caveolin-3, fibroblasts were labeled with vimentin, and endothelial cells were labeled with isolectin. Nuclei were labeled with Hoechst 33342. Scale bar = 50 μm. Images previously published in [28]. C, transverse section of 300 μm rat myocardial slice with ≈ 15 layers of cardiomyocytes visualized and native architecture maintained. Sarcomeric structures labeled with α-actinin, sarcolemma labeled with WGA (wheat germ agglutinin), nuclei labeled with Hoechst 33342. Image acquired with immunohistochemical staining and confocal microscopy by Ifigeneia Bardi

## Bridging the Gap Between Cardiovascular Models

Basic cardiac research has been traditionally performed on biological models ranging from isolated cardiomyocytes to complex multicellular preparations. Isolated cardiomyocytes have provided the bedrock for research over the last few decades. However, during enzymatic isolation, connections between neighboring cardiac cells and the extracellular matrix are lost and a high proportion of cardiomyocytes is damaged, with significant implications for cardiomyocyte function [2–5]. Multicellular research platforms, including isolated whole hearts and intact tissue preparations (isolated trabeculae/perfused tissue wedges), avoid the issues associated with cell isolation, but their use is typically limited to acute time points due to issues associated with ex vivo perfusion and tissue ischemia [6]. A preparation that bridges the gap between overly simplified isolated cardiomyocyte studies and whole-heart studies, where it is difficult to study changes at the cellular/molecular level, is still a major unmet need in cardiac research.

The ultrathin nature of myocardial slices combines the benefits of a complex multicellular three-dimensional preparation with the simplicity of acquiring structural and functional data from a two-dimensional monolayer. Myocardial slices have allowed our group to study different cardiac cell populations within their physiological location in a representative manner avoiding enzymatic isolation [7]. The maximum tissue diffusion distance for oxygen is ≈ 200 μm [8]. As myocardial slices have a thickness of 300 μm, the innermost cells are a maximum of 150 μm from either slice surface and small molecules have been shown to rapidly diffuse through the full tissue thickness [9], making them more amenable to chronic culture. Additionally, our laboratory has developed several assays that allow the real-time detection of changes in cardiac structure and function with high temporal and spatial resolution, which is feasible with whole-heart or in vivo studies. These include the detection of average sarcomere length within myocardial slices using laser diffraction, which can be carried out alongside functional assessment of contractility or Ca^2+^ handling. A major advantage provided by myocardial slices is that several slices can be prepared from each heart/specimen, increasing experimental throughput while also reducing the number of animals used in research. Finally and most importantly, from a translational perspective, myocardial slices can be produced from human biopsies [1, 10]. Many translational approaches are severely hampered by the profound disparities between animal and human physiology and myocardial slices are capable of providing a clinically relevant human in vitro model system. Cardiac tissue wedges, trabeculae and Langendorff-perfused whole hearts are the only other multicellular preparations that can be utilized from human specimens. The extremely limited availability of whole human hearts and the technical challenges of maintaining these preparations in vitro make myocardial slices the only human multicellular platform with preserved structure and function, and with the possibility for chronic culture and in vitro manipulation.

## Development and Challenges of Preparation

There has been a slow but progressive increase in the number of laboratories preparing myocardial slices since they were first described [11]. Myocardial slices have been utilized for several studies, including biochemistry, metabolism, electrophysiology, and pharmacological cardiotoxicity studies [12–15]. Additionally, techniques have been developed to preserve the viability of myocardial slices for several days in vitro [16]. In recent years, interest has grown as the advantages provided by myocardial slices for translational research have been more widely realized. However, a major obstacle to the more widespread use of myocardial slices has been the lack of a consensus regarding optimal preparation. This has resulted in substantial variability in methods used and varying myocardial slice quality between laboratories. In light of this, our laboratory systematically analyzed the method by which both we and others prepared myocardial slices. This highlighted several fundamental differences between protocols, including the method by which hearts were explanted, different techniques for isolating and mounting ventricular tissue blocks, and the use of different settings and conditions for slicing cardiac tissue.

## A Novel Protocol and Improved Outcomes

**Fig. 2:**
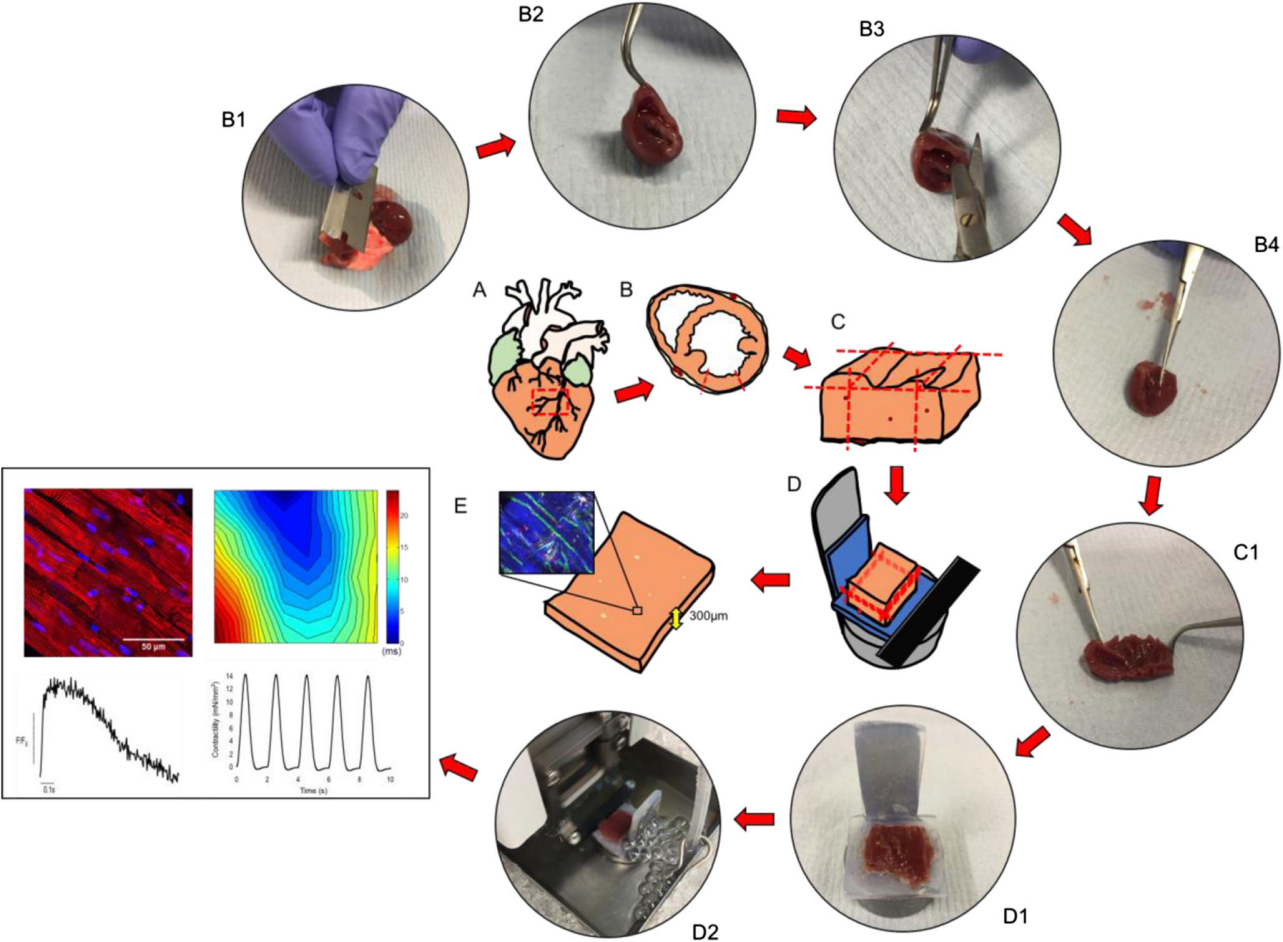
Preparation of myocardial slices. A, small or large mammalian hearts were explanted. B, the left ventricle was then isolated from the whole heart; B1, the lungs and atria were removed by making an incision through the ventricular base; B2 and B3, the right ventricle was dissected off; B4, an incision was made along the septum towards the apex to open the left ventricle. C, left ventricular tissue block was prepared; C1, papillary muscle was dissected to ensure the tissue block was as flat as possible. D, tissue block was mounted and sliced using a high precision vibratome; D1, tissue block was mounted epicardium down to ensure it was as flat as possible; D2, tissue block was sliced while submerged in ice-cold Tyrode’s solution + 2,3-butanedione monoxime while vigorously bubbled with 100% O_2_. E, 300 μm myocardial slices were used for structural studies (red, alpha-actinin; blue, Hoechst 33342) and functional studies (conduction velocity with multielectrode array, Ca^2+^ handling with Fluo-4 and optical mapping, contractility recording using a force transducer). Images in this figure have been previously published in *Nature Protocols* and *Cardiovascular Research* [1, 7]

In the optimized protocol that our group published in *Nature Protocols* [1], we described several fundamental steps that were vital to ensure minimal tissue damage during slice preparation. The first issue was the method by which hearts were explanted (Fig. 2A). We determined that the method employed should depend on the size of the heart being explanted. While it was possible to explant small mammalian (mice and rats), hearts directly following sacrifice with the heart still beating, this approach was not appropriate for larger mammals. Larger hearts, with thicker ventricular walls, required in situ cold cardioplegic arrest prior to explantation to provide adequate cardioprotection and minimize loss of viability. Following explantation, ventricular tissue blocks were isolated and mounted on the vibratome specimen holder. Several laboratories embed the tissue block in low melting point agarose before proceeding with the cutting. In the optimized protocol published in *Nature Protocols* [1], we do not advocate this practice as this approach is not necessary during the slicing process and may further reduce tissue oxygenation during slicing, resulting in ischemic damage. A very important factor we identified, which had been often overlooked, was the identification of myocardial fiber orientation and fiber alignment within tissue blocks. In a large number of studies, hearts were sliced in the short-axis or embedded in agar with little regard for fiber orientation [9, 17–21]. These approaches frequently resulted in substantial numbers of myocardial fibers being transected, resulting in a higher proportion of dead and damaged cardiomyocytes, poor quality structural/functional data, and substantial variability. Histological studies have demonstrated that myocardial fibers within the left ventricle are arranged into discrete sheets, which have an ordered laminar structure and are orientated transversally to the ventricular wall [22]. In our protocol, we described a method for dissecting the left ventricle of small and large mammalian hearts to ensure tissue flattening. When combined with mounting of the tissue block epicardium-down, a previously described technique we optimized [13], sheets of myocardial fibers are aligned in the same plane of the vibratome blade, ensuring minimal tissue damage during slicing. This is particularly important when working with small mammalian hearts, which have a more pronounced ventricular curvature. The use of an excitation-contraction uncoupler (2,3-butanedione monoxime), optimal vibratome settings (vibratome z-axis error < 1 μm), temperature control (~ 4 °C), and careful tissue handling techniques were also fundamental. Using this approach, damage incurred during slicing is strictly limited to the myocardial slice surface, with only ≈ 2–3% of the total cardiomyocyte population damaged during slicing. The improvement in the quality of myocardial slices produced also allowed us to visualize myocardial fiber orientation within each slice using a macroscope. We were then able to dissect out areas (≈ 8 × 8mm) where myocardial fibers were highly aligned, further improving the quality by reducing areas with damaged cardiomyocytes and consequently reducing the variability of data acquired. Using this refined and optimized approach, we were able to significantly improve our contractility measurements and now obtain slices producing ~ 15 mN/mm^2^ [1].

## Applications of Myocardial Slices

**Fig. 3:**
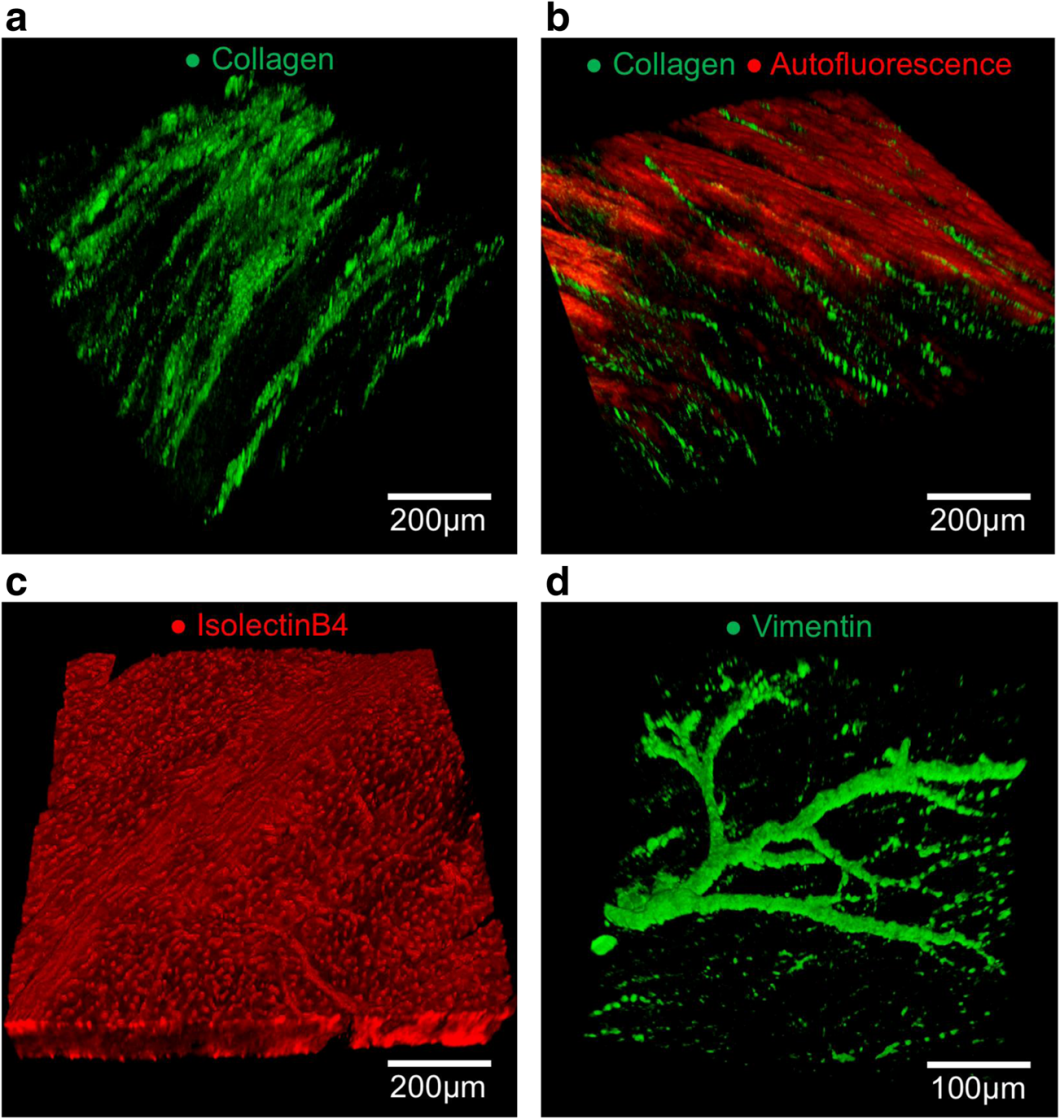
Analysis of collagen distribution and vascular networks using a FASTClear protocol. A, second-harmonic generation imaging of collagen type 1 in a human heart failure myocardial slice. B, autofluorescence is used to visualize the structure of the myocardium of a healthy dog myocardial slice. Collagen is arranged into stands between the myocardial fibers. C, three-dimensional reconstruction of healthy dog myocardial slice microvasculature stained with isolectinB4. D, three-dimensional reconstruction of healthy dog myocardial slice microvasculature stained with vimentin. Images in this figure have been previously published in *Scientific Reports* [25]

As myocardial slices provide a clinically relevant and representative human model system in vitro, they are particularly useful for translational studies. A substantial amount of research has already demonstrated that myocardial slices are an appropriate model for acute pharmacological testing and in vitro safety screening [10, 12, 13, 23] particularly as they have been found to have more representative electrophysiological properties than other cardiac models [14]. A number of early cell-based regenerative medicine strategies were also explored using this platform [18, 20, 24]. Our laboratory is now using myocardial slices to determine the effects and regenerative potential of state-of-the-art engineered heart tissue technologies on the heart. In addition, we have developed a rapid and versatile Free-of-Acrylamide SDS-based Tissue Clearing (FASTClear) protocol specifically for myocardial slices. This technique facilitated the analysis of collagen content, localization, and three-dimensional distribution through the full thickness of pathological human and healthy dog myocardial slices (300 μm) (Fig. 3A/B) and is a useful tool for studying cardiac fibrosis. The protocol does not induce structural alterations and can be combined with immunohistochemical staining to image microvascular and macrovascular networks [25] (Fig. 3C/D). This provides significant advantages over conventional imaging techniques, which are typically limited to surface structures. Myocardial slices have provided a useful platform for the development of conductive biomaterials [26, 27], which are capable of modulating cardiac conduction velocity. The data acquired were similar to those obtained from ex vivo Langendorff perfused hearts, suggesting slices can be a more high-throughput and affordable alternative for these types of studies. Finally, myocardial slices provide an optimal in vitro platform for studying many novel translational research themes, including cardiac reprogramming, and there is significant interest from the pharmaceutical and biotechnology sectors for this growing technology.

## A Perfect Model for Chronic In Vitro Studies?

Techniques have been developed to culture myocardial slices in vitro. The current gold standard methodology is based on the protocol described by Brandenburger et al [16]. In brief, myocardial slices are cultured at a liquid-air interface using semi-porous tissue culture inserts which are placed in 6-well plates. The tissue slices have access to both culture medium on one side and oxygen on the other side which allows cell survival for up to 28 days. Although this methodology is simple and inexpensive, myocardial slices undergo a process of substantial dedifferentiation, which results in proliferation of stromal cells and in the loss of the sophisticated ultrastructure and function that characterizes adult cardiomyocytes [7]. While myocardial slices remain viable, their use as a representative model remains debatable, which has limited current studies to acute time points. What is required is a new technique for culturing myocardial slices in their native state, with preserved structure and function, for several days. Our laboratory and others are focused on this issue and substantial progress is being made. We recently described a method for culturing myocardial slices with a static mechanical load using A-frame stretchers, which significantly attenuated the proliferation of cardiac fibroblasts on cultured slices at 3 and 7 days [7]. Further work is still required to develop techniques to prevent the progressive loss of other structural and functional parameters during culture. The prolonged maintenance of myocardial slices in culture is likely to open new and exciting avenues in cardiovascular research, including pharmacological testing and the development of novel therapeutic strategies.
